# The genetic basis and potential molecular mechanism of yellow-albino northern snakehead (*Channa argus*)

**DOI:** 10.1098/rsob.220235

**Published:** 2023-02-15

**Authors:** Donglei Sun, Xin Qi, Haishen Wen, Chao Li, Jianlong Li, Jiwei Chen, Zexin Tao, Mingxin Zhu, Xiaoyan Zhang, Yun Li

**Affiliations:** ^1^ Key Laboratory of Mariculture (Ocean University of China), Ministry of Education (KLMME), Fisheries College, Ocean University of China, Qingdao 266003, People's Republic of China; ^2^ School of Marine Science and Engineering, Qingdao Agricultural University, Qingdao 266109, People's Republic of China

**Keywords:** northern snakehead, albinism, whole-genome sequencing, RNA-Seq, knockdown and rescue experiments, *slc45a2*

## Abstract

Body colour is an important economic trait for commercial fishes. Recently, a new colour morph displaying market-favoured yellow skin (termed as yellow-mutant, YM) of northern snakehead (*Channa argus*) was discovered in China. We confirmed that YM snakehead is an albino with complete loss of melanin in the skin and eyes by histological and ultrastructural observations, and inherited as a recessive Mendelian trait. By applying genomic analysis approaches, in combination with gene knockdown and rescue experiments, we suggested a non-sense mutation in *slc45a2* (c.383G > A) is the causation for the YM snakehead. Notably, significantly higher levels of key melanogenesis genes (*tyr*, *tyrp1*, *dct* and *pmel*) and phospho-MITF protein were detected in YM snakehead than those in wild-type individuals, and the underlying mechanism was further investigated by comparative transcriptomic analysis. Results revealed that differential expressed genes involved in pathways like MAPK, WNT and calcium signalling were significantly induced in YM snakehead, which might account for the increased amount of melanogenesis elements, and presumably be stimulated by fibroblast-derived melanogenic factors in a paracrine manner. Our study clarified the genetic basis of colour variation in *C. argus* and provided the preliminary clue indicating the potential involvement of fibroblasts in pigmentation in fish.

## Graphical abstract

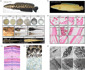

## 1. Introduction

**Figure 1.**
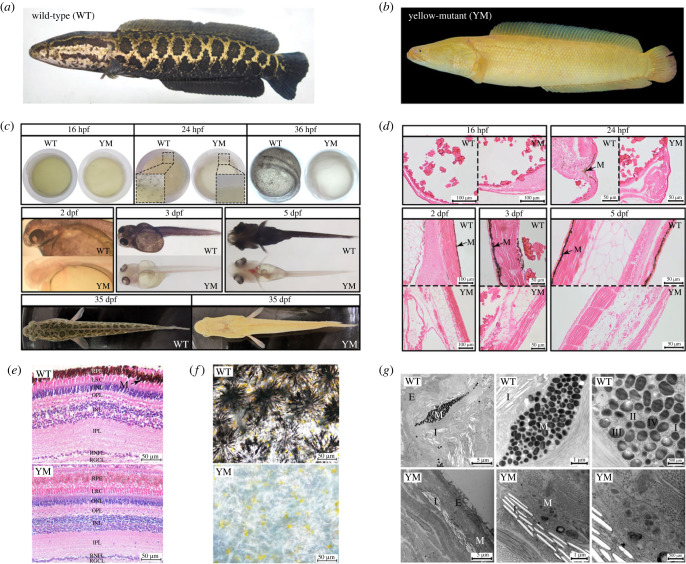
The comparative observation of pigmentation phenotype between YM and WT snakeheads. (*a*) The snakehead adult displaying WT phenotype*.* (*b*) The snakehead adult displaying YM phenotype. (*c*) Morphologic observation on embryonic and larvae of WT and YM snakeheads. (*d*) Histological observation on embryonic and larvae of WT and YM snakeheads. Longitudinal sections show the dorsal sides of 16–24 hpf embryos and 2–5 dpf larvae, respectively. (*e*) Histological observation of juvenile fish eyes in WT and YM snakeheads. RPE: retinal pigment epithelium; LRC: layer of rods and cones; ONL: outer nuclear layer; OPL: outer plexus layer; INL: internal nuclear layer; IPL: inner plexus layer; RNFL: retinal nerve fibre layer; RGCL: retinal ganglion cell layer. (*f*) Microscopic observation of juvenile fish skins in WT and YM snakeheads. (*g*) Ultrastructure observation of juvenile fish skins in WT and YM snakeheads. hpf: hours post-fertilization, dpf: days post-fertilization. M: melanophore; I: iridophore; E: epithelium. I, II, III, IV: stages of melanosome development.

The northern snakehead (*Channa argus*) is an economically important freshwater species being cultured widely in Asia and Africa [1]. With its fast growth, high nutrition value, significant hypoxia-resistance capacity and pharmaceutical properties usage in Chinese traditional medicine [2], *C. argus* has become extremely popular in China with the annual production exceeding 501 095 tons [3]. During the breeding of *C. argus*, three varieties with distinct colour morphs have been discovered in Chinese aquaculture industry, including the wild-type (WT) with greyish-black body colour (figure 1*a*), the widely reported white-albino strain with stable inheritance of the white skin [4] and the newly discovered strain with the golden yellow skin (figure 1*b*). The golden yellow snakehead was originally discovered in Shandong Province (35.78° N, 118.62° E), showing the loss of melanin pigmentation on the body surface and retina (hereafter named as yellow-mutant (YM) snakehead). So far, as the golden yellow body pigmentation is more attractive to consumers especially for aquarium fans, the market price for the YM snakehead is significantly higher than that for the WT. However, the mechanism for the cause of yellow-albino morph remains elusive.

Albinism is characterized by complete or nearly complete absence of melanin in the eyes and the body [5,6], which is frequently observed in a variety of vertebrates including mammals [7,8], reptiles [9,10], amphibians [11,12], birds [13,14] and fishes [15,16]. Mammals and birds possess only one type of pigment cell, melanocyte, which can produce two different types of melanin, namely eumelanin (brown/black) and pheomelanin (yellow/red). By contrast, at least six pigment cell types known as chromatophores, including melanophore (black), xanthophore (yellow), erythrophore (red), iridophore (iridescent), leucophore (whitish) and cyanophore (blue), have been identified in fish species [17]. Among them, fish melanophores are structurally and functionally similar to melanocytes, which produce only eumelanin, but no pheomelanin [18]. Nonetheless, the biosynthesis and regulation pathways for the black pigment are conserved to a certain extent in widely divergent vertebrate taxa.

Melanin is synthesized in the specific lysosome-related organelles termed melanosomes within melanophores/melanocytes [19]. Melanosomes undergo four distinct stages depending on the degree of maturation (stages I–IV) [20]. During these stages, well-established factors related to melanin production can be categorized into three groups, including structural proteins required for melanosomes (PMEL/gp100, MART-1 and GPNMB), enzymatic proteins involved in melanogenesis (TYR, TYRP1 and TYRP2/DCT), and proteins regulating trafficking and processing (APs, OA1, BLOC-1, P-protein and Rab27a) [21]. In addition, several transcription factors (CREB, MITF and PAX3) and G protein-coupled receptor (MC1R) are also involved in regulating melanocyte/melanophore functions [21–24]. Therefore, a number of genes have been identified to regulate melanin production at different levels, and mutation in many of them may affect coat colour, or give rise to albinism in animals [22]. For example, in human, mutations in *TYR*, *OCA2*, *TYRP1*, *SLC24A5* or *SLC45A2* lead to oculocutaneous albinism, and mutations in genes related to cellular functions have been identified as responsible for causing ocular albinism (*GPR143*, *GNAI3*), Hermansky-Pudlak syndrome (*BLOC1-3*, *AP-3*) or Chediak-Higashi syndrome (*LYST*, *CHS1*) [25,26]. Pigment-related gene mutations have also been identified in albino mice [27,28], monkey [29], horse [30], dog [31] and chicken [32]. Albino phenotypes are more frequently observed in fishes [33,34] and usually considered as an important appearance trait producing higher economic and ornamental values than the wild-type for aquaculture species; however, the causative genes of colour mutation have not been clarified in most of them.

In recent years, the rapid progress in the development of high-throughput sequencing technologies has provided valuable genomic tools for discovering the genetic contributors for albino phenotypes in teleosts. For example, the genome sequencing and analysis of strain-specific variants revealed that mutations in *oca2* are likely to be the cause of albinism in albino goldfish strains [35], and also as the reason for the amelanistic morph of Malawi golden cichlid fish [36]. The genome-wide association study (GWAS) analysis indicated *Hps4* as the candidate gene of the catfish albinism [16]. Additionally, bulked segregant analysis (BSA) demonstrated that mutations in *Hps5* may induce oculocutaneous albinism in three-spine stickleback [37]. Nevertheless, the causality of the effects of candidate genes has not been verified in most of the aquaculture fishes due to practical limitations.

In this study, we used genomic resources including whole-genome resequencing and transcriptomic data, in combination with phenotypic, genetic and functional assays, which were integrated to clarify the genetic basis and molecular regulatory mechanism of the albinism in YM snakehead. Our findings not only illuminated the molecular basis underlying the body colour variation of *C. argus*, but also provided insight to the molecular mechanism of albinism in fish.

## 2. Results and discussion

### 2.1. YM snakehead displays complete loss of melanin in the skin and eyes

YM snakehead possesses higher market value than WT because of its attractive skin coloration, which could bring considerable economic benefits to the *C. argus* industry. To examine whether yellow pigment phenotype is albinism, we investigated the status of melanin synthesis by histological and ultrastructural observations. As results showed, for the WT snakehead, the neural crest (NC)-derived body melanophores became visible at 24 h post-fertilization (hpf), while the retinal pigment epithelium started melanization before 2 days post-fertilization (dpf) (figure 1*c*). Correspondingly, histological sections revealed that the NC-derived pigment cells firstly appeared under the epidermal layer of the embryo of WT snakehead at 24 hpf, with the melanin content increased significantly at hatching time (2 dpf) and post-hatch stages (3–5 dpf) (figure 1*d*). By contrast, for the YM, melanin in NC-derived melanophores was undetectable across the body during the entire tested periods (figure 1*c*). It seemed to present slight pigmentation in the eyes of YM snakehead, which was proved not to be melanin through histological observation (figure 1*e*), as well as homogeneous eye tissues visualized in the tubes (electronic supplementary material, figure S1). The colour may result from the microvessels in the iris.

Direct observation through microscope for fresh skin tissue of juvenile fish showed that WT snakehead contained the mature dendritic melanophores with typical melanin granules and the round xanthophores with yellow pigments (such as pteridines and carotenoids), whereas only xanthophores were detected in YM snakehead (figure 1*f*). Further, the ultrastructural morphology of juvenile skin showed that WT skin contained large melanosomes in all stages of maturation, primarily of stage III and stage IV, while YM melanophores displayed fewer, smaller and immature melanosomes (figure 1*g*). In addition, fishes' iridophores usually contain large, thin and flat-shape purine crystals called reflecting platelets [18]; however, only numerous cavities were detected in both WT and YM (figure 1*g*), which may result from loss of reflecting platelet crystals from prepared sections as glutaraldehyde fixation or dehydration through an alcohol or acetone series [18]. Our results demonstrated that the complete loss of melanin in YM snakehead is the direct cause of the yellow colour phenotype.

### 2.2. YM snakehead is inherited in autosomal-recessive manner

**Figure 2.**
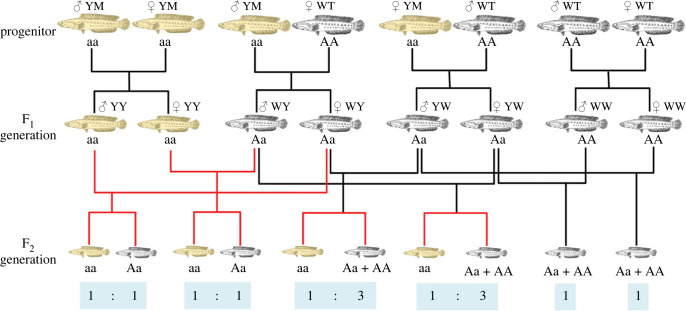
Pedigree analysis of crosses between YM snakeheads, between WT snakeheads, and reciprocal crosses between YM and WT snakeheads. The ratio of snakeheads displaying the yellow and WT pigmentations in the F_2_ generation is shown at the bottom.

The inheritance pattern of albinism has been studied earlier in a variety of aquaculture fishes. In several investigated cases, the albinism was controlled by an autosomal-recessive mutation, such as grass carp [5] and channel catfish [16,38]. On the contrary, the red body colour of tilapia is dominant controlled by a single gene [39]. In the case of rainbow trout, both monogenic recessive and dominant albino mutations have been described [40–42]. For other species like *Cyprinus carpio*, the pigment-related traits were identified to be determined by polygenic factors [43,44]. To test the inherent pattern of body coloration in YM snakehead, different crosses were constructed to produce F_1_ and F_2_ generation progenies to investigate segregation. Results show that all F_1_ offspring derived from the crosses within WT or YM snakehead resembled the body colour of their parents, which were black (100%) for WW, and yellow (100%) for YY (figure 2; electronic supplementary material, table S2). Meanwhile, all F_1_ offspring from reciprocal crosses between WT and YM snakehead (WY and YM) showed black phenotype (100%), indicating the black colour of WT snakehead is dominant over the yellow of mutants (figure 2). Further, the observed frequencies of F_2_ generation approximated to the typical Mendelian segregation ratio of 1 : 1 for offspring generated from YW♀ × YY♂ (0.95 : 1), YY♀ × YW♂ (1.05 : 1), WY♀ × YY♂ (1.07 : 1) and YY♀ × WY♂ (0.95 : 1), as well as 3 : 1 for offspring generated from YW♀ × YW♂ (3.08 : 1), WY♀ × WY♂ (2.86 : 1), WY♀ × YW♂ (2.91 : 1) and YW♀ × WY♂ (3.05 : 1), respectively (figure 2; electronic supplementary material, table S2), demonstrating that the yellow colour mutant in *C. argus* was inherited in autosomal-recessive manner.

### 2.3. Identification of the candidate causal genomic locus for the YM phenotype by screening single-nucleotide polymorphism markers

**Figure 3.**
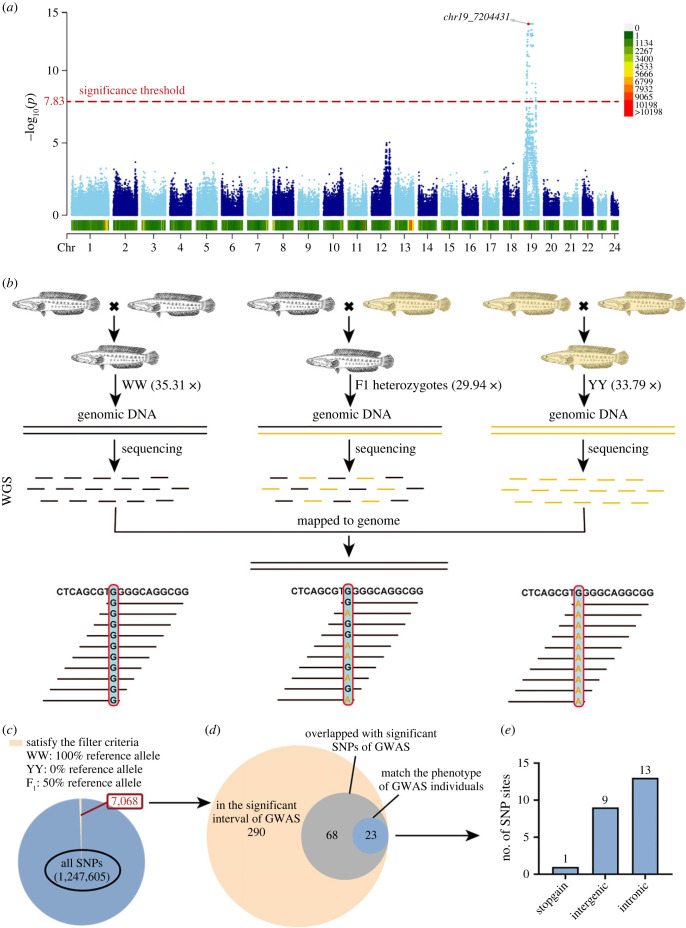
Genomic analysis process of identification of the genomic loci associated with the pigmentation phenotype of YM snakehead*.* (*a*) GWAS analysis using logistic regression modelling revealed that only one genomic region (13.98 Mb) at Chr19 was responsible for the pigmentation phenotype of YM snakehead. (*b*) Identification of allele frequency differences by WGS method among three DNA bulks, generated from homozygous WW pool, YY pool and F_1_ heterozygotes pool, respectively. (*c*) A total of 1 247 605 SNPs were detected among the three WGS bulks. SNP variants were further screened, resulting in 7068 qualified SNPs passing all filtering criteria. (*d*) Two hundred and ninety fell into the 13.98 Mb genomic interval of GWAS, containing 68 SNPs overlapped with the significantly associated SNPs of GWAS in 13.98 Mb candidate genomic interval. (*e*) A total of 23 filtered SNPs that were perfectly associated with pigmentation phenotype of the 40 fish samples used in GWAS analysis, which contained 1 non-sense (stop-gain) variant, 9 intergenic variants and 13 intronic variants.

We sequenced the whole genomes of 20 yellow-albino phenotype and 20 black phenotype individuals of the F_2_ progeny, which were generated from intercross between YW♀ and WY♂, producing 9.26 Gb whole-genome sequencing (WGS) data per fish on average (approx. 12.91×) (electronic supplementary material, table S3). After filtering, a total of 671 874 high-quality single-nucleotide polymorphisms (SNPs) was obtained for GWAS using the logistic regression model, which revealed a single genome-wide peak (genome-wide significance threshold was calculated as 7.83), a 13.98 Mb genomic region on Chromosome 19 (4 542 570–18 525 309), containing 1017 significantly associated SNPs within this region (figure 3*a*).

To further narrow down the candidate genomic loci, we performed WGS on unrelated fish individuals, generating three DNA bulks with homozygous WW pool (*n* = 24, coverage = 35.31×) and YY pool (*n* = 24, coverage = 33.79×), as well as F_1_ heterozygous pool (*n* = 24, coverage = 29.94×), respectively, to measure the allele frequency differences (electronic supplementary material, table S3). Because the yellow phenotype is controlled by the recessive factor, SNPs were screened by allele genotype according to the following criteria: (i) SNPs must consist of only two allele types (one for the reference allele, another for variant allele); (ii) SNP sites at WW (same as the reference allele) and YY (the variant allele) are homozygous with different genotypes, but are heterozygous at F_1_ heterozygotes (contained both reference allele and variant allele). The Chi-square goodness-of-fit with a null hypothesis of equal amounts of the two alleles (with the 1 : 1 ratio) in the F_1_ heterozygotes was used to examine the allele ratio. After screening, 7068 SNPs passed all filtering criteria, of which 290 fell into the 13.98 Mb candidate genomic interval of GWAS. Among them, 68 SNPs were overlapped with the significantly associated SNPs of GWAS, containing 23 SNPs that were perfectly associated with pigmentation trait of fish samples used in GWAS analysis, which means all 20 yellow-albino individuals possessed ‘aa’, and all 20 individuals with black phenotype have ‘AA’ or ‘Aa’ at these SNP loci (figure 3*b–d*). Only one of these SNPs was located in the protein-coding region, resulting in non-sense mutation in the gene of *slc45a2* (figure 3*e*).

### 2.4. A premature stop codon in SLC45A2 might be responsible for YM phenotype

**Figure 4.**
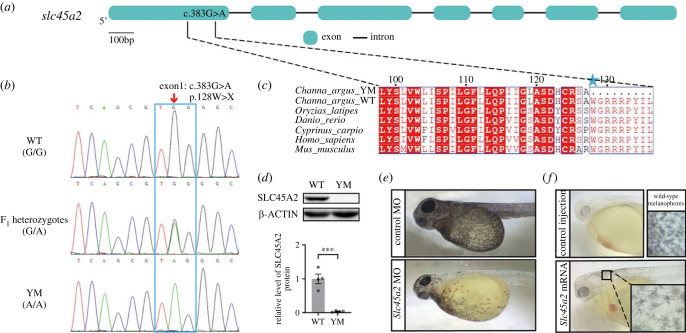
The causative mutation in SLC45A2 of YM snakehead. (*a*) Schematic diagram showing the mutation site of *slc45a2* was located at the exon1 (c.383G > A) in YM snakehead. (*b*) Sanger sequencing validation for the candidate mutation site of *slc45a2* in WT, YM and F_1_ heterozygotes. (*c*) Multiple alignment of SLC45A2 amino acid sequences among vertebrates. The site of residue W128 of SLC45A2 indicated by the blue pentagram was highly conserved among selected species. (*d*) Protein levels of SLC45A2 in WT and YM snakehead skins were detected by western blotting. Relative expression levels of SLC45A2 protein are shown as the mean ± s.e.m. (*n* = 4), *** *p* < 0.001. The western blots are cropped and blot source data are available in the electronic supplementary material, figure S2. (*e*) Morpholino-mediated gene knockdown of *slc45a2* reduces the melanin content severely in WT snakehead. (*f*) mRNA injection of *slc45a2* rescues the melanin production in YM snakehead*.* MO: morpholino.

Based on WGS analysis, we identified the non-sense mutation of *slc45a2* as the candidate causative mutation for YM phenotype (figure 4*a*). Correspondingly, *SLC45A2* gene has been identified to be responsible for the dilute phenotype in the cream horse [30], silver chicken [32] and white tiger [7], as well as the pale orange skin of medaka mutant strain [45]. In humans, SLC45A2 mutations cause oculocutaneous albinism type 4 (OCA4), which is an autosomal-recessive disorder with abnormal melanin formation in the skin, hair follicles and eyes [46].

We next validated the identified mutation site in *slc45a2* via Sanger sequencing using a number of samples of WT, YM and F_1_ heterozygote individuals, and the results were consistent with expected segregation pattern (figure 4*b*). As shown in figure 4*a*, the mutation site in YM snakehead was located at the nucleotide position 383 (c.383G > A) of *slc45a2*. The G-to-A transition in the first exon led to a stop codon TAG that substituted the tryptophan at the residue 128 and resulted in the translation termination. The site of residue W128 of SLC45A2 was found to be highly conserved among vertebrates (figure 4*c*). The early stop codon in SLC45A2 was predicted to truncate the protein in the major facilitator superfamily homologue domain [47], likely resulting in non-functional protein. Afterwards, we examined the protein level of SLC45A2. As expected, due to the premature stop codon, the protein of SLC45A2 was not detected in YM skin, whereas abundant SLC45A2 protein was found in WT individuals (figure 4*d*).

To confirm the function of *slc45a2* for melanin synthesis in *C. argus*, the ATG-blocking morpholino designed against *slc45a2* was injected into the yolk at 1–4 cell stages of WT snakehead embryos. About 24 h after injection, 34 WT embryos injected with *slc45a2* morpholino displayed a severe reduction of pigmentation, and the melanin distributed unevenly (figure 4*e*). Alternatively, the controls injected with standard control morpholino retained the normal pigmentation, with the dense melanin uniformly distributed over the whole skin (figure 4*e*).

Furthermore, the mRNA rescue experiment was performed to determine if exogenous WT *slc45a2* mRNA could rescue the albino phenotype. As shown in figure 4*f*, injection of *slc45a2^WT^* significantly increased pigmentation levels in YM embryos, several dendritic melanophores being observed at the NC. In total, 52 larvae displayed different degree of pigmentation recovery in YM fish. On the contrary, injection of physiological saline into the YM embryos did not lead to any phenotypic changes (figure 4*f*). Therefore, morpholino-based knockdown and mRNA rescue experiments suggested strongly that the non-sense mutation in *slc45a2* was responsible for the albino phenotype of YM snakehead and we refer to this allele as *slc45a2^YM^*.

SLC45A2 was recognized as a H^+^-coupled sugar cotransporter, containing 12 transmembrane domains [48]. Two independent roles of SLC45A2 in melanogenesis have been reported: to promote TYR processing and intracellular trafficking to melanosomes, and to positively regulate pH neutralization and TYR function through H^+^ efflux [24]. Both explanations have been supported by experimental evidences [49–51], but the function of SLC45A2 in pH regulation is more certain. Melanosomes in different stages have different internal pH [24]. Non-pigmented melanosomes of stage I–II exhibit acidic pH, which are generated by vacuolar H^+^-ATPase (V-ATPase) through mediating H^+^ influx [52]. Stage III–IV melanosomes are modulated to neutral pH, in order to preserve the function of TYR, the rate-limiting enzyme in the process of melanin production [24,53]. The internal pH of melanosomes is neutralized by a combinational function of several membrane transporters, of which SLC45A2 is suggested to be the most promising molecule by several lines of evidence [24,54]. For examples, the functional deficiency of TYR in *slc45a2*-mutant albino zebrafish can be rescued by inhibition of V-ATPase or SLC45A2 RNA injection [50]. Also, the knockdown of SLC45A2 in human melanoma cell line reduced melanosomal pH, tyrosinase activity and melanin content [55]. Recently, it was demonstrated that Slc45a2 deficiency significantly increased the acidification of melanosomal pH through enhanced glycolysis to inhibit melanin biosynthesis and promote melanoma metastasis [54].

### 2.5. The expressions of core melanogenesis genes and nuclear phospho-MITF protein level significantly increased in YM snakehead

**Figure 5.**
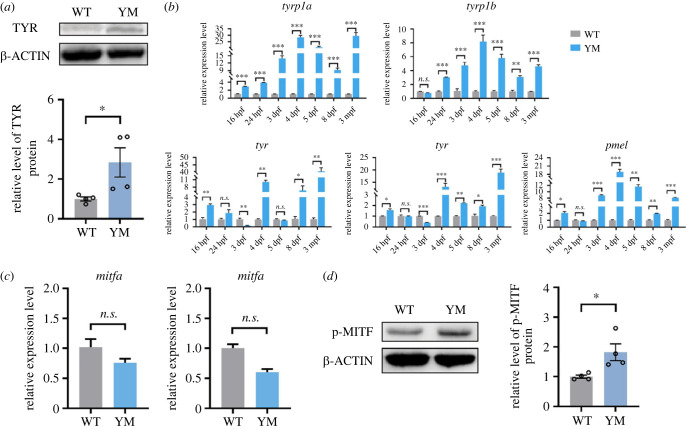
The comparison of core melanogenesis genes in skins between WT and YM snakeheads. (*a*) Western blotting detection of TYR protein levels in skins of WT and YM snakeheads (mean ± s.e.m., *n* = 4). (*b*) qPCR detection of mRNA levels of the melanogenic genes (*tyr*, *tyrp1a*, *tyrp1b*, *dct* and *pmel*) at different ontogenetic stages (mean ± s.e.m., *n* = 3). (*c*) qPCR detection of mRNA levels of *mitfa* and *mitfb* in skins of WT and YM snakeheads (mean ± s.e.m., *n* = 3). (*d*) Western blotting detection of phospho-MITF(Ser180) protein levels in skins of WT and YM snakeheads (mean ± s.e.m., *n* = 4). The western blots are cropped and blot source data are available in the electronic supplementary material, figures S3 and S4. * *p* < 0.05, ** *p* < 0.01, *** *p* < 0.001, *n.s*.: not significant.

We conducted western blotting to detect weather the protein level of TYR was affected by the loss-of-function mutation of SLC45A2 in YM snakehead. Notably results showed that the TYR protein level in skins of YM was significantly higher than that of the WT (figure 5*a*). We then performed quantitative polymerase chain reaction (qPCR) to detect the mRNA expression levels of *tyr* gene, as well as other melanogenic genes which are known to play key functions in tyrosine metabolism including tyrosinase genes (*tyrp1a*, *tyrp1b*, *dct* and *pmel*) [53]. Corresponding to upregulated protein level of TYR, all of these genes exhibited significant higher expression levels in YM than in WT snakehead (figure 5*b*). Additionally, the expression values of all five genes in YM were dramatically higher than those in WT during different ontogenetic stages (16 hpf, 24 hpf, 3 dpf, 4 dpf, 5 dpf, 8 dpf and 3 mpf) (figure 5*b*).

We subsequently detected the expression level of microphthalmia-associated transcription factor (MITF), which is the master transcription factor that regulates melanogenesis by activating the transcription of downstream melanogenic genes including *tyr*, *tyrp1*, *dct* and *pmel* [53,56]. Although the two coding gene copies of *mitf* (*mitfa* and *mitfb*) did not show significant expression difference between the two skin types at the transcriptional level (figure 5*c*), we demonstrated that the nuclear phospho-MITF(Ser180) protein level in YM group was significantly higher than that in WT group by western blotting (figure 5*d*), which should be the reason for the upregulated expression of the above-mentioned melanogenic genes [57–60].

### 2.6. RNA-Seq analysis provided preliminary clues for revealing the potential regulatory mechanism of melanogenesis in snakeheads

**Figure 6.**
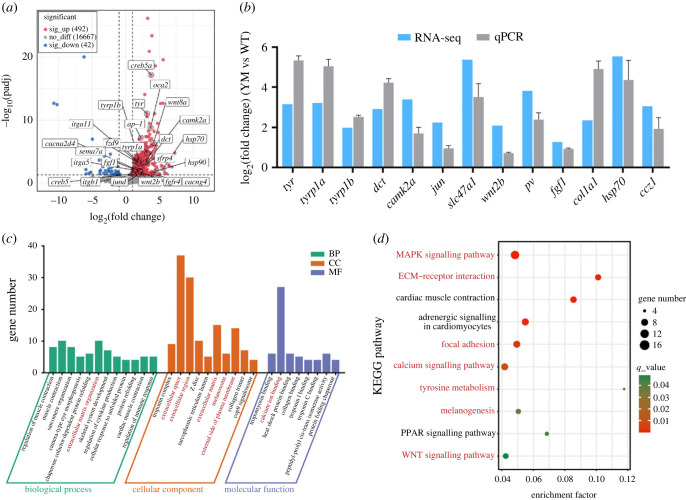
The DEGs between skins of WT and YM snakeheads and enrichment analysis. (*a*) Volcano plot showing DEGs between skins of WT and YM snakeheads. Red dots and blue dots indicate the significantly upregulated and downregulated genes in YM snakeheads, respectively. Grey dots indicate genes that were not expressed differently between the two groups. The candidate DEGs involved in the regulation of melanogenesis, melanosome biogenesis, melanosome transport, tyrosine metabolism and fibroblast-derived melanogenic factors are marked in the figure. (*b*) Validation of RNA-Seq analysis results by qPCR (*n* = 3). (*c*) GO enrichment analysis for DEGs. Bar graph description of top 30 enriched GO terms of DEGs between skins of WT and YM snakeheads. (*d*) KEGG pathway enrichment analysis for DEGs. Scatter diagram of the significantly enriched KEGG pathways for DEGs between skins of WT and YM snakeheads (*q*-value < 0.05). The colour and size of dots indicate the *q*-value and the number of DEGs assigned to the corresponding pathway, respectively.

To further investigate the molecular changes and potential regulatory pathways in the control of melanogenesis underlying the loss-of-function mutation of SLC45A2, RNA-Seq analysis was performed on the skin tissues of WT and YM snakehead to compare the gene expression differences. We obtained a total of 492 324 374 raw reads from six cDNA libraries, of which 484 226 220 (about 72.63 Gb) clean reads remained for further analysis after discarding the adapter, poly-N or low-quality sequences. The mapped clean reads percentage of the six cDNA libraries ranged from 91.83% to 94.60%. The average clean Q30 and GC percentage for each library was greater than 92.37% and 45.62%, respectively (electronic supplementary material, table S4), which indicated that our RNA-Seq data were of high quality and suitable for the subsequent analyses. A total of 534 differential expressed genes (DEGs) were identified using DESeq2 software (*p*-adjusted < 0.05, |log_2_(fold change)| < 1), of which 492 genes were upregulated and 42 were downregulated in the YM snakehead compared with the WT snakehead, with the log_2_(fold change) values ranging from 7.28 to −10.69 (figure 6*a*; electronic supplementary material, table S5). To validate the RNA-Seq results, we selected 13 genes involved in melanin biosynthesis for qPCR analysis. As shown in figure 6*b*, the qPCR expression patterns of 13 DEGs agreed with the results of RNA-Seq analysis.

All DEGs generated from RNA-Seq data were subjected to GO and KEGG functional enrichment analysis. Results showed that the most concentrated GO terms of DEGs between WT and YM individuals contained extracellular matrix (ECM) organization (GO:0030198) in biological process (BP), extracellular space (GO:0005615), extracellular region (GO:0005576), ECM (GO:0031012), external side of plasma membrane (GO:0009897) and melanosome (GO:0042470) in cell composition, as well as calcium ion binding (GO: 0005509) in molecular function (figure 6*c*). KEGG enrichment analysis revealed that besides pigmentation-related pathways like tyrosine metabolism and melanogenesis, DEGs were significantly enriched in several signal transduction and interaction pathways like MAPK, WNT and calcium signalling pathways, as well as ECM–receptor interaction and focal adhesion pathways (figure 6*d*; electronic supplementary material, table S6). We identified 38 candidate DEGs genes involved in the regulation of melanogenesis, melanosome biogenesis, melanosome transport, tyrosine metabolism and ECM molecules (electronic supplementary material, table S6). Those DEGs in most of above-enriched GO terms and KEGG pathways were significantly upregulated in the skin of YM snakehead (electronic supplementary material, table S6), which may arise from the feedback on melanogenesis due to the lack of melanin protection against UV radiation from sunlight [61–65].

**Figure 7.**
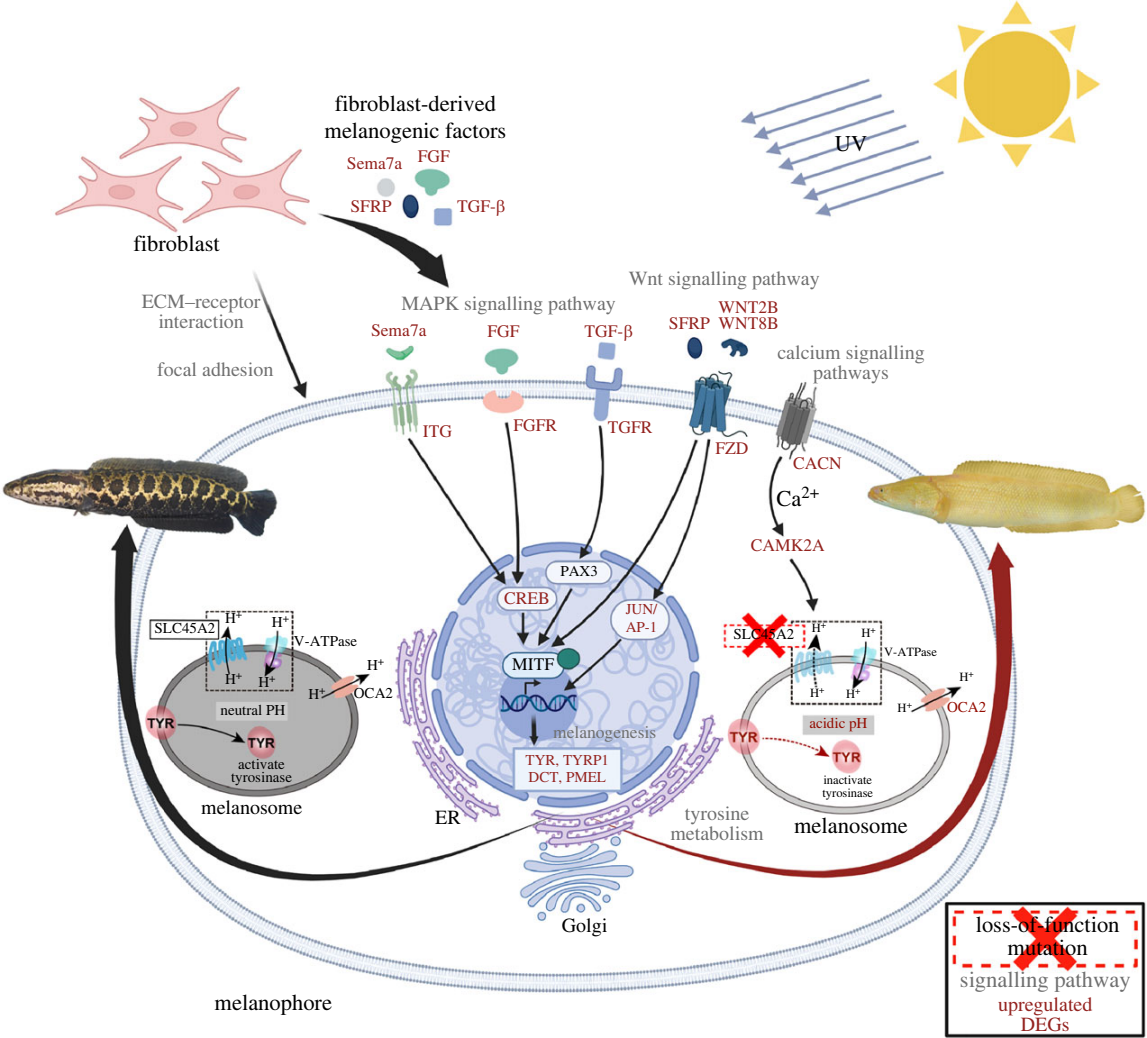
Schematic diagram reveals predicted molecular mechanism regulating the response to the loss-of-function mutation of SLC45A2 in *C. argus*. The mutation of SLC45A2 induces melanosomal acidification, leading to the inactivation of TYR and therefore the failure to synthesize melanin in YM snakehead*.* Due to the lack of melanin, UV irradiation penetrating through the epidermis increases dramatically and triggers the dermal fibroblast-derived melanogenic factors (TGF-β, FGF, SFRP and Sema7a), which act on the melanophore by binding to receptors and modulating intracellular signalling cascades (MAPK, WNT and calcium). The induced signalling pathways elevate the phosphorylation of MITF, thereafter upregulating the downstream key melanogenesis genes (*tyr*, *tyrp1, dct* and *pmel*). Red font indicates the significantly upregulated DEGs. The full names of these genes are listed in the electronic supplementary material, table S6.

Based on the above bioinformatic analysis results in combination with manual literature searches, we speculated the potential functions of DEGs and enriched pathways in pigmentation underlying the YM phenotype. Our hypothesis is summarized in figure 7 and discussed as follows. (i) At the skin of YM snakehead, we detected an increase in phosphorylated MITF protein level, leading to the upregulated expressions of downstream melanogenesis genes such as *tyr*, *tyrp1*,* dct* and *pmel*. However, this did not result in a higher synthesis of melanin due to the loss-of-function mutation of SLC45A2, which might cause failure to neutralize the melanosomal pH and inactivate TYR [24]. (ii) The increased amount of p-MITF protein in YM snakehead was suggested to be regulated by the induced DEGs involved in signalling pathways like MAPK, WNT and calcium, which have been widely demonstrated as crucial pathways regulating melanin synthesis [64,65]. (iii) These intracellular signalling pathways might be stimulated by mediators from neighbour cells, such as fibroblasts, since we have identified several fibroblast-derived melanogenic factors showing increased expression in YM snakehead (figure 7; electronic supplementary material, tables S5 and S6). Although this has not been proved in fish species, studies of mammals have increasingly demonstrated the important role of fibroblasts in the process of skin pigmentation [66]. These studies indicated that melanogenic factors were synthesized by fibroblasts and function in a paracrine manner on melanocytes [66]. In the case of YM snakehead, we speculated that the lack of melanin leads to reduced UV absorption and increased UV penetration through the epidermis [67], which might be the trigger for the upregulations of these dermal fibroblast-derived melanogenic factors, as well as above-mentioned melanogenic related genes and pathways. However, our assumption about the roles of fibroblasts in fish pigmentation needs to be further validated by functional experiments.

## 3. Conclusion

In this study, we characterized the newly discovered colour morph of northern snakehead possessing pleasant golden yellow skin being an albino with complete loss of melanin, inherited in an autosomal-recessive manner. The genetic basis of body colour mutation was investigated by WGS approaches in combination with gene knockdown and rescue experiments, which indicated that a premature stop codon in *slc45a2* was the causative mutation. We surprisingly found an elevated phospho-MITF protein level and key melanogenesis genes in the YM snakehead, which was speculated to be stimulated by fibroblast-derived melanogenic factors deduced from RNA-Seq results. Our study has elucidated the underlying molecular basis for body colour variation in YM snakehead, which has already been explored as a target for new variety breeding programme in China. Additionally, the precise role of fibroblasts in melanophore pigmentation needs to be investigated in future studies.

## 4. Methods

### 4.1. Chromatophore examination, histological and ultrastructural observation

The experimental fish were reared in a *C. argus* breeding farm named Daqiang Fishery Co. Ltd in Linyi, Shandong Province, China (35.78° N, 118.62° E). All the experimental fish were raised in outside ponds. Embryos and larvae of WT and YM snakehead at different developmental stages were collected. Half of these samples were used for direct morphologic observation, and the other half were fixed for 24 h in Bouin's solution for histology. Juveniles (body length: 17.6 ± 1.8 cm; body weight: 78.1 ± 12.5 g) of two colour morphs were anaesthetized with tricaine methane sulfonate (MS-222) in a dose of 300 ppm before sampling. Subsequently, eyes were dissected, and part of them were fixed in Bouin's solution, meanwhile the other part was placed in 1.5 ml microfuge tubes, and homogenized in 1 ml of phosphate-buffered saline (PBS) buffer by a homogenizer (DHS Life Science & Technology Co. Ltd, China). Small pieces of skin at the dorsal side above the lateral line were surgically excised (electronic supplementary material, figure S5) and washed with PBS for direct observation. In addition, the same position of skin tissues was collected for histological and ultrastructural observation. Those fixed samples were dehydrated through a graded alcohol series and embedded in paraffin wax, sectioned at 6 µm using an RM 2016 microtome (Leica, Germany) and stained with eosin. Five sections of each sample were taken for observation and photography. All the above images were visualized and captured by a DP73 microscope digital camera (Olympus, Japan).

Ultrathin sections for ultrastructural observation were prepared as follows: skin tissue was fixed in 0.1 M PBS (pH 7.4) containing 2.5% glutaraldehyde for 12 h, washed for 30 min in PBS, post-fixed for 1 h in 1% osmium tetroxide and then dehydrated through an ascending series of ethanol and embedded in epoxide resin. Subsequently, sections of 80 nm were obtained using an ultramicrotome and were mounted on multiple-hole copper grids, stained with uranyl acetate and lead citrate. Twenty ultrathin sections were obtained from each of WT and YM skin samples. Five images were taken from each section. These images were examined using a transmission electron microscope (JEM-1200EX, Japan) operating at 80 kV. Four distinct stages of melanosomes depending on the degree of maturation were defined. In detail, stage I melanosomes are unpigmented spherical vacuoles with an amorphous matrix; stage II melanosomes exhibit an ellipsoidal shape containing a fibrillar internal matrix; stage III melanosomes contain a structured matrix with apparent melanin deposition; finally, the melanosomes are fully pigmented with highly dense melanin deposits in stage IV [19,68].

### 4.2. Construction of WT and YM snakehead crosses

*Channa argus* founders from the WT and YM variants were selected for artificial reproduction, following the method of Kahkesh [69] with minor modifications. A total of eight F_1_ families were established by crossing between two colour morphs or within the same colour type (electronic supplementary material, figure S6). In detail, three F_1_ families were generated by crossing between WT females and YM males (WT♀ × YM♂, termed WY), three reciprocal F_1_ families were constructed by mating YM females with WT males (YM♀ × WT♂, YW), and one family for WT (WT♀ × WT♂, WW) and one for YM type (YM♀ × YM♂, YY) were established.

After 2 years of cultivation, well-developed F_1_ individuals reaching sexual maturity were selected from each family to produce F_2_ generation. The mating pairs were settled as follows: 1# (YW♀ × YW♂), 2# (WY♀ × WY♂), 3# (YW♀ × WY♂), 4# (WY♀ × YW♂), 5# (YW♀ × YY♂), 6# (YY♀ × YW♂), 7# (WY♀ × YY♂), 8# (YY♀ × WY♂), 9# (YY♂ × WW♀) and 10# (YY♀ × WW♂) (electronic supplementary material, figure S6). The segregation ratios of the offspring for each mating pair were counted by random sampling for three times with about 500 offspring per time.

### 4.3. Whole-genome resequencing and genome-wide association analysis

Twenty fish individuals with each of yellow phenotype and black phenotype were randomly selected from the 3# (YW♀ × WY♂) family of the F_2_ generation. These 40 fish were used for GWAS to identify candidate genomic loci responsible for body colour mutations. For each sample, genomic DNA was extracted from fin tissue using the TIANamp Marine Animals DNA Kit (TIANGEN, China). The 150 bp paired-end sequencing libraries with insert size of 350 bp were constructed following the instruction of TruSeq Library Construction Kit (Illumina, USA) and then were sequenced by DNBSEQ-T7 platform.

Raw reads generated from WGS were processed to remove the low-quality sequences by fastp (v 0.20.0) with default parameters, and the clean reads of all libraries were separately mapped to the reference genome of the *C. argus* (accession no. JAJQTP000000000) using BWA (v 0.7.17) mem mode (settings: mem -t 4 -k 32 -M -R) [70]. Alignment files were converted to BAM files using SAMtools (v 1.10) (settings: -bS -t) [71]. In addition, potential PCR duplications were removed using SAMtools command ‘rmdup’. Variants calling were performed for all BAM files by the HaplotypeCaller protocol in Genome Analysis Toolkit (GATK, v 3.8) (http://www.broadinstitute.org/gatk/download) [72]. The SNPs were filtered using GATK VariantFiltration with parameter setting as follows: --filterExpression ‘QD < 4.0, FS > 60.0, MQ < 40.0’, -G_filter ‘GQ < 20’. InDel was filtered by the following parameters: --filter Expression ‘QD < 4.0, FS > 200.0’. We further filtered the variants with ‘--min-meanDP 5, --max-meanDP 250, --max-missing 0.95’ using VCFtools (v 0.1.16) [73]. Logistic regression model using Plink (v 1.90) software [74] was used to perform the GWAS analysis. The statistical significance threshold was defined by a Bonferroni correction for multiple testing (−log_10_(0.01/*N*), where *N* is the number of total SNPs used for association test). The CMplot R package was used to create the Manhattan plot (https://github.com/yinlilin/cmplot). The significant SNPs were annotated by ANNOVAR [75] based on the GFF files of the reference genome.

To further verify the genomic loci potentially linked to pigmentation genes, three WGS libraries including WW pool, YY pool and F_1_ heterozygotes pool were generated using fish samples of F_1_ families, and were used for BSA analysis. For each library, genomic DNA was extracted from fin tissue samples of 24 fish individuals and equally pooled. One hundred and fifty paired-end sequencing was performed on the Illumina Hiseq™ platform. Sequencing data processing and SNP filtering were performed using the same method as the above GWAS analysis.

### 4.4. RNA-Seq analysis

For transcriptome sequencing, skin tissues from 9 WT and 9 YM snakehead were extracted using TRIzol (Invitrogen, USA). Equal amounts of RNA from three individuals for each colour variant were pooled, and a total of six sequencing libraries were generated using NEBNext® Ultra™ RNA Library Prep Kit for Illumina® (NEB, USA) following the manufacturer's instructions. Then, the libraries were sequenced on an Illumina HiseqTM platform for generating 150 bp paired-end reads.

The raw sequencing data were subjected to adaptor trimming and quality filtering using Trimmomatic (v. 0.39). The obtained clean reads were aligned to the reference genome of *C. argus* using Hisat2 (v. 2.0.4). The gene expression levels were normalized by the FPKM algorithm. Transcripts were considered to be expressed when the FPKM values > 1 in the three biological replicates. Differential gene expression analysis was performed using the DESeq2 R package (v 1.0). The DEGs were determined with the thresholds of *q*-value (adjusted *p*-value using the Benjamini and Hochberg method) < 0.05 and |log_2_(fold change)| > 1. Enrichment analyses based on GO and KEGG were further performed to identify the function of the DEGs.

### 4.5. Sanger sequencing validation for candidate mutation site

The mutation site of *slc45a2* was tested in WT, YM and F_1_ heterozygotes (WY or YW). Thirty individuals were selected from each population for DNA extraction and used as templates for PCR reaction, and these fish were unrelated individuals. The PCR reaction system contained 1 µl each of forward and reverse primers (10 pM) for *slc45a2* gene (forward: 5′- TGACCCTGTTATCAGAGGACCAG-3′; reverse: 5′-CTGAGATGATAGCATCCCCGTT-3′), 10 µl of Taq PCR Mix 2× (Vazyme, China), 1 µl of DNA template and 7 µl RNAase-free water. PCR amplification was performed on a T100™ Thermal Cycler (Bio-Rad, Germany) as follows: initial denaturation at 95°C for 3 min, followed by 35 cycles at 95°C for 30 s, 55–60°C for 30 s and 72°C for 10 min. PCR products with expected sizes were sequenced by the Sanger sequencing method (BGI company, China).

### 4.6. Quantitative real-time polymerase chain reaction

The qPCR was performed to validate the results of RNA-Seq, while the relative gene expression levels of selected genes (*tyr*, *tyrp1a*, *tyrp1b*, *dct*, *camk2a*, *jun*, *slc47a1*, *wnt2b*, *pv*, *fgf1*, *col1a1*, *hsp70* and *ccz1*) were detected in adult skin tissues of the two colour variants. In addition, the expression levels of key genes for melanin synthesis including *tyr*, *tyrp1a*, *tyrp1b*, *dct*, *pmel* were examined in different developmental stages. The primer sequences are listed in electronic supplementary material, table S1. The qPCR reaction volume was 20 µl, including 10 µl of SYBR®FAST qPCR Master Mix (2×), 0.4 µl ROX reference dye, 0.4 µl each of forward/reverse primers (10 pM), 6.8 µl RNAase-free water and 2.0 µl of cDNA template (10× diluted). qPCR was performed using a StepOne Plus Real-Time PCR system (Applied Biosystems, USA) and was run in accordance with following procedure: 95°C for 30 s, followed by 40 cycles of 95°C for 5 s, 60°C for 30 s and a final extension at 72°C for 2 min. The *β-actin* were used as internal reference genes to normalize the gene expression level and the relative gene expression levels were calculated using the 2^−ΔΔCT^ method.

### 4.7. Morpholino-mediated gene knockdown

Using the full-length sequence of *C. argus slc45a2*, a morpholino was designed to the 5′ ATG translational start site. Both the ATG-blocking morpholino targeting *slc45a2*, and the standard control morpholino were obtained from Gene Tools (https://www.gene-tools.com/). The sequences were designed as follows: *slc45a2*_atg: 5′-GTCCTCTGATAACAGGGTCATGGT-3′ and standard control: 5′-CCTCTTACCTCAGTTACAATTTATA-3′. A mixture containing 1 mM morpholino and 1.0% phenol red indicator was injected into the yolk at 1–4 cell stage embryos of WT snakehead with a PCO-1500 Microinjector (ZGENEBIO, China). After injection, embryos were incubated at 26°C until hatching. The phenotype of injected embryos was observed from 24 hpf (when the melanin began to appear) and photographed by an SZ810 stereomicroscope (CNOPTEC, China).

### 4.8. Rescue experiment by messenger RNA injection

The full-length sequence of *slc45a2* was amplified from cDNA derived from WT *C. argus* via PCR using the following primers: *slc45a2-*HindIII F: 5′-ctagcgtttaaacttaagcttATGACCCTGTTATCAGAGGACCA-3′ and *slc45a2-*BamHI R: 5′- ccacactggactagtggatccTCAATCCACATATCTGACAAAGAGG-3′. The product was ligated into the pcDNA3.1 (+) vector using the ClonExpress® Ultra One Step Cloning Kit (Vazyme, China). The resulting plasmid was verified by sequencing, and messenger RNA was transcribed using the T7 High Yield RNA Transcription Kit (Vazyme, China). Injections were carried out at 1–4 cell stage of YM snakehead at a concentration of 200 ng µl^−1^. After injection, embryos were incubated at 26°C until hatching. The phenotype of injected embryos was observed from 24 hpf and photographed by an SZ810 stereomicroscope (CNOPTEC, China).

### 4.9. Western blot analysis

Western blotting assay was carried out to compare protein abundances of TYR and SLC45A2, and phosphorylation levels of MITF between WT and YM snakehead. In detail, RIPA lysis buffer (Solarbio, China) containing 1% phenylmethylsulfonyl fluoride was applied to isolate total protein from skin tissues. Then, the supernatant was harvested following centrifugation at 4°C for 5 min at a speed of 12 000 rpm, and the protein concentrations were assessed with a BCA Protein Quantification Kit (Vazyme, China). After that, proteins were denatured at 98°C for 8 min and an equal amount of proteins from each sample were separated by 10% SDS-PAGE and then transferred into 0.25 µm PVDF membranes (Millipore, Billerica, MA, USA). The membranes were blocked using QuickBlock blocking buffer (Beyotime, China) for 2 h and incubated with the indicated primary antibodies overnight at 4°C. The primary antibodies included (i) the rabbit anti-human primary antibody TYR (1 : 500, No. ab180753, Abcam, UK), (ii) the rabbit anti-*C. argus* primary antibody SLC45A2 obtained from the anti-serum of immunized rabbits which has been injected by the synthesized SLC45A2 peptide (EVQQKPRGSNESLRG) of *C. argus* (Genecreate, China), (iii) the phospho-specific primary antibody for MITF (p-MITF (Ser180) antibody, No. AF3027, Affinity Biosciences, China) and (iv) the rat anti-human primary antibody β-ACTIN (1 : 1000, Beyotime, China). TBST was washed three times, then HRP-labelled goat anti-rabbit and goat anti-rat secondary antibody (1 : 5000, Sangon Biotech, China) was added and incubated at 37°C for 1 h. TBST was washed three times. Colour was developed with ECL chemiluminescence solution, protein expression signalling was detected by using a gel imaging analysis system (Eberhardzell, Germany). ImageJ software was applied for protein quantitation. All experiments were performed in triplicate.

### 4.10. Statistical analysis

Gene expression and statistical analysis were carried out as described above. All experiments were carried out in triplicate. Values are presented as the mean ± s.e.m. Statistical analysis was performed by GraphPad Prism 8.0 software (GraphPad Software Inc., USA). Univariate analysis of variance was performed using SPSS 25.0 software (IBM, USA), and *p*-values < 0.05 were considered statistically significant.

## Data Availability

The whole-genome resequencing data have been deposited in the NCBI Sequence Read Archive (SRA) with accession no. PRJNA834927. The transcriptome data can be accessed with accession no. PRJNA772548. The data are provided in the electronic supplementary material [76].
